# Privacy–Accuracy Consideration in Devices That Collect Sensor-Based Information

**DOI:** 10.3390/s21144684

**Published:** 2021-07-09

**Authors:** Lihi Dery, Artyom Jelnov

**Affiliations:** 1 Department of Industrial Engineering and Management, Ariel University, Ariel 40700, Israel; 2 Ariel Cyber Innovation Center, Ariel University, Ariel 40700, Israel; 3 Economics and Business, Ariel University, Ariel 40700, Israel; artyomj@ariel.ac.il

**Keywords:** user–device interaction, privacy, smart devices, sensor-based information, privacy–accuracy trade-off

## Abstract

Accurately tailored support such as advice or assistance can increase user satisfaction from interactions with smart devices; however, in order to achieve high accuracy, the device must obtain and exploit private user data and thus confidential user information might be jeopardized. We provide an analysis of this privacy–accuracy trade-off. We assume two positive correlations: a user’s utility from a device is positively correlated with the user’s privacy risk and also with the quality of the advice or assistance offered by the device. The extent of the privacy risk is unknown to the user. Thus, privacy concerned users might choose not to interact with devices they deem as unsafe. We suggest that at the first period of usage, the device should choose not to employ the full capability of its advice or assistance capabilities, since this may intimidate users from adopting it. Using three analytical propositions, we further offer an optimal policy for smart device exploitation of private data for the purpose of interactions with users.

## 1. Introduction

Personal data can improve user experience as users receive personally tailored advice or assistance. For example, smart watch devices that track user movements suggest exercise when they detect the user is inactive for a long period of time [1]. Similarly, infrared sensors can detect falls and call for assistance [2]. However, in order to advise or assist, devices require access to personal information and these data require protection.

It is often unclear to the user what private information is collected, and if and how it is protected. Citing from a survey on the economics of privacy of [3]: “[Users’] ability to make informed decisions about their privacy is severely hindered because consumers are often in a position of imperfect or asymmetric information regarding when their data are collected, for what purposes, and with what consequences”.

The ability of individuals to manage privacy amid increasingly complex trade-offs is a problem, as faulty decisions may lead to privacy violations, which in turn incur various consequences. First, if information about users is leaked, it enables price discrimination. A second aspect is that of the violation of the user’s right to “peace and quiet”; for example, when receiving undesired adware (see [4]). Lastly, privacy violations might make it possible to sell user information to a third party.

We herein consider devices that employ user private information collected from sensors or devices provided with sensors. For example, by wearing a smart watch, the user shares information such as his location, heartbeat and movements. Using this information, the device is able to produce more accurate services. Users may be oblivious to the privacy risk at first. After an initial period in which they use the device, and once the device collects the data that are used to offer advice or assistance, the users may become aware of the potential privacy risk. The users must trust the device to continue using it once they are aware of the privacy risk. When the device is not trusted, privacy risks and privacy violations may lead to users abandoning the device (also known as customer churn [5]). User perception of privacy and trust are therefore important [6,7,8].

**Contributions:** In this paper, we provide an analytical examination of how users’ considerations of privacy risk affect their interactions with sensors and devices provided with sensors. We propose a model that shows that the user–device interaction has an interesting trend for users moderately concerned about their privacy. Nothing the device suggests will impact the very concerned or the very oblivious users, since they either do not trust the device with their information or are not concerned at all. However, the device’s usage by users with a moderate privacy concern depends on how the users perceive the device’s privacy risk. When the risk is not completely known to the users upfront, the users will use the device only if they trust it. With moderately concerned users, the device should choose not to employ the full capability of its advice or assistance capabilities, since this may intimidate the user from using it. Our results are not limited to any specific device and are generally true for any sensor or device provided with sensors that utilizes information where user privacy might be compromised.

The rest of the paper proceeds as follows. We begin with some background (Section 2). We then present a game-theoretical model and analytical results (Section 3). As some of the model is without mathematical proofs, we provide a numerical solution to the model (Section 4). We conclude with a discussion of our main findings (Section 5).

## 2. Related Work

As Acquisti et al. [3] have stated, “privacy is difficult to define”. In a similar manner, we focus on the informational dimension of privacy. i.e., the protection or sharing of personal data. There are two attitudes to the protection of private data: the protection is either handled by the device or by the user. When the privacy is left in the hands of the users, it is known as “privacy self-management” [9]. In some cases, install-time permissions provide users with control over their privacy as users are required to decide to whom to provide consent to collect, use and disclose their personal data [10]. In other cases, the users can choose between public and personal operation mode, or switch between these modes according to their activity context [11]. Often, users require assistance in privacy-related decisions [12].

Considerable research has focused on privacy from the device side, i.e., a technical perspective such as securing the channels over which information is sent [13] or collected [14]. Some researchers suggest zero-touch non-invasive systems where users do not need to engage with the system [15], while others secure the privacy of by-standers [16]. Interviews surveying how users perceive privacy of wearable devices conclude that there are a variety of user attitudes ranging from users who are not worried at all to users that are highly concerned for their privacy [17]. However, these studies do not consider actions following what users deem as a privacy risk nor do they present recommendations for devices.

John et al. [18] have experimentally shown that privacy-related cues affect the extent to which users are concerned about their privacy. Accordingly, previous research has emphasized the role of the clarity of the privacy policies on the user trust in the device or system used; changing the look of privacy policies makes online services appear more trustworthy [19]. Deciding which IOT-related devices are appropriate depends on the user familiarity more than it does on the privacy policy [20]. Similarly, [21] analyze the effects of both cognitive trust and emotional trust on the intention to opt in to health information exchanges and willingness to disclose health information.

We study scenarios where the users implicitly deduce the extent to which their private information is being analyzed, from the behavior of the device, as displayed in the advice or assistance the device offers. A user with a new smart watch might not bother to read the privacy policy of the smart watch’s app. Nevertheless, after a short period of time, the user can easily deduce that they are being monitored, for example, when the watch suggests the user should stretch, exercise or even breathe deeply [22]. It has been experimentally shown that smart watch usage is directly influenced by perceived usefulness and perceived privacy risk has a direct negative influence on the behavioral intention to use smart watches [23,24,25].

The above studies are experimental. For a general game theoretic model, where players exchange some information while being concerned about privacy, see [26] for example. In a more related context, Jullien et al. [27] discuss website users in situations where a website sells user information to third parties, which may lead to a good, a bad or a neutral experience for the users. In these situations, user vulnerability to a bad experience is unknown to the website. They consider a framework with two periods, where the users decide whether to stay with the website for the second period, depending on the first period outcome. In this work we implement the same two-period framework. However, Jullien et al. [27] study vulnerability as a property of the users, while we suggest examining risk as a property of the device.

In sum, previous studies that have focused on trust and privacy have shown that both have a direct effect on the usage of devices. However, these studies are of an experimental nature. In this paper we present a complementary analytical model that can explain the experimental results others have collected, support their claims and provide a better understanding of the privacy–accuracy trade-off for smart and sensor-based devices.

## 3. Model

We herein employ a game theoretic approach and examine a model containing a device with various possible degrees of privacy risks, and users that are uncertain as to the device privacy risk. In order to achieve high accuracy the device must obtain and exploit private user data and thus confidential user information might be jeopardized. We define accuracy as the degree of closeness of the advice or assistance offered by the device to the advice and assistance the user actually requires. We provide an analysis of this privacy–accuracy trade-off. We assume that a user’s utility from a device is positively correlated with:

1. The user’s privacy risk.
2. The advice or assistance offered by the device.

We consider an initial stage where the device only collects data, and a continuous stage where the device exploits the collected data.

For simplicity, we define two degrees of risks: (1) High risk, meaning that the user data are public or might be shared or sold to third parties and (2) Low risk, meaning that the user data are confidential. Let there be a user (*C*) and a device (*S*). The device has a high privacy risk (*H*) with probability π and a low privacy risk (*L*) with probability 1−π. We denote the device type by ρ={H,L}. This ρ is unknown to the users. For convenience, all of the notation are found in Table 1.

Before usage, the user activates the device. Thus the user receives an initial signal of the device type, which is correct with probability 1−ϵ and erroneous with probability ϵ. Namely, if ρ=H, the user receives a signal *h* with probability 1−ϵ and a signal *l* with probability ϵ. If ρ=L, the user receives a signal *l* with probability 1−ϵ and a signal *h* with probability ϵ.

At **stage 1** (initial usage), the device chooses the accuracy level of its support algorithm (advice and/or assistance) q∈[0,1]. We denote by $q_{H}$ and $q_{L}$ strategies chosen by the high type and the low type devices, respectively. The user receives adjusted support with probability $P_{a}\left(q,\rho \right)$. That is, the device’s support depends on the algorithm’s level of accuracy, and on the extent to which the device utilizes the private information it collected from the user. With a high risk device, the private information is more likely to be utilized, and vice versa. We assume that $P_{a}\left(q,\rho \right)$ increases in *q* and for every *q*, $P_{a}\left(q,L\right)<P_{a}\left(q,H\right)$. Denote by *a* the event “support is sent to the user”, and by $\bar{a}$ the complementary event. Following *a*, the user’s utility is $u_{1}>0$ and device’s utility is $v_{1}>0$. For $\bar{a}$, both the user and the device obtain utility 0. Following signal s∈{h,l} and event $e\in \{a,\bar{a}\}$, the user assigns a probability to the device being H: P(H|s,e)

At **stage 2** (continuous usage), the user decides whether to keep using the device or to limit, reduce or abandon the device altogether. For simplicity, we look at two options: *leave* or *not leave*.

If the user leaves, both user and device obtain a utility of 0. If the user does not leave, with probability $P_{b}\left(\rho \right)$ (ρ is S’s type), the user’s private information is leaked, exposing the user to possible damage. Let $P_{b}\left(H\right)>P_{b}\left(L\right)$. The utilities of the user and the device in this case are $u_{b}<0$ and $v_{b}$, respectively. With probability $1-P_{b}\left(\rho \right)$, the user uses the device, and his/her utility is $u_{H}$ and $u_{L}$, if ρ=H or ρ=L, respectively. We assume that $u_{H}>u_{L}$, namely, that the high type device has more value to the user, but this type has a higher privacy risk. We also assume that expected utilities of both *H* and *L* in stage 2 (denoted as $E_{H}^{2}$ and $E_{L}^{2}$, respectively) are positive. The user’s total utility is a total sum of the outcomes of stages 1 and 2.

We now turn to analyze if and when the users will abandon the device. If the potential damage $|u_{b}|$ to the user is high, the user will leave the device, regardless of their belief of the device’s type. The opposite is also true. If $|u_{b}|$ is low, they will not leave regardless of their belief of the device type. For intermediate values of $|u_{b}|$, the user’s strategy depends on the utility $u_{H}$ of leaving a high-type device, when no damage is caused. If $u_{H}$ is high, the user does not leave if they assign a sufficiently high probability to be a high-type device; however, if the utility $u_{H}$ is relatively low, the user does not leave only if they assign a sufficiently low probability to the device being high type. Recall that the risk of being damaged by not abandoning the device is higher if the device type is H. This analysis is formally stated in the following proposition. The proofs of all propositions appear in the Appendix A.

**Proposition** **1.** *Consider a Nash pure strategy equilibrium.*

*1.*  *Let $u_{H}>\frac{P_{b}\left(H\right)u_{L}\left(1-P_{b}\left(L\right)\right)}{\left(1-P_{b}\left(H\right)\right)P_{b}\left(L\right)}$. Then in Nash equilibrium:*
  *(a)*  *If $\frac{\left(1-P_{b}\left(H\right)\right)u_{H}}{P_{b}\left(H\right)}<\left|u_{b}\right|$, C prefers to leave at stage 2 for any P(H|s,e).*
  *(b)*  *If $|u_{b}|<\frac{\left(1-P_{b}\left(L\right)\right)u_{L}}{P_{b}\left(L\right)}$, C prefers not to leave at stage 2 for any P(H|s,e).*
  *(c)*  If $\frac{\left(1-P_{b}\left(L\right)\right)u_{L}}{P_{b}\left(L\right)}<\left|u_{b}\right|<\frac{\left(1-P_{b}\left(H\right)\right)u_{H}}{P_{b}\left(H\right)}$, there exists $P_{H}^{*}$ such that C chooses not to leave iff $P_{H}^{*}<P\left(H|s,e\right)$.
*2.*  *Let $u_{H}<\frac{P_{b}\left(H\right)u_{L}\left(1-P_{b}\left(L\right)\right)}{\left(1-P_{b}\left(H\right)\right)P_{b}\left(L\right)}$. Then in Nash equilibrium:*
  *(a)*  *If $|u_{b}|<\frac{\left(1-P_{b}\left(H\right)\right)u_{H}}{P_{b}\left(H\right)}$, C prefers not to leave at stage 2 for any P(H|s,e).*
  *(b)*  *If $\frac{\left(1-P_{b}\left(L\right)\right)u_{L}}{P_{b}\left(L\right)}<\left|u_{b}\right|$, C prefers to leave at stage 2 for any P(H|s,e).*
  *(c)*  If $\frac{\left(1-P_{b}\left(H\right)\right)u_{H}}{P_{b}\left(H\right)}<\left|u_{b}\right|<\frac{\left(1-P_{b}\left(L\right)\right)u_{L}}{P_{b}\left(L\right)}$, there exists $P_{H}^{*}$ such that C chooses not to leave iff $P\left(H|s,e\right)<P_{H}^{*}$.

The next proposition states that at stage 1, if the signal about the device type is noisy (high ϵ), the device may choose not to offer maximal quality support ($q_{H}=q_{L}=1$). The reason being that when the quality is maximal, the probability of tailored support increases, and thus, the user’s belief that the device type is *H* increases. At stage 2, if the benefit of *H* is sufficiently low (a low $u_{H}$), the user may leave the device after receiving the tailored support.

**Proposition** **2.** *Suppose $v_{1}<E_{H}^{2}$ and $v_{1}<E_{L}^{2}$. Let $u_{H}<\frac{P_{b}\left(H\right)u_{L}\left(1-P_{b}\left(L\right)\right)}{\left(1-P_{b}\left(H\right)\right)P_{b}\left(L\right)}$. Assume $P_{a}\left(1,H\right)\left[1-P_{a}\left(1,L\right)\right]>P_{a}\left(1,L\right)\left[1-P_{a}\left(1,H\right)\right]$. Then there is $\epsilon <\frac{1}{2}$ and $u_{b}$ such that $q_{H}=q_{L}=1$ is not a Nash equilibrium strategy of H and L.*

When the signal about the device type is sufficiently precise, the user knows the device type with a high probability. Therefore, the user chooses whether to leave or not at stage 2 regardless of the outcomes of stage 1. In this case, the device chooses maximal quality (i.e., the best tailored support). Formally:

**Proposition** **3.** For each $u_{b}<0$, there is $\epsilon^{*}>0$ such that for all $\epsilon <\epsilon^{*}$, $q_{H}=q_{L}=1$ is a unique equilibrium.

## 4. Numerical Results

We present the following results using Monte Carlo simulations and computed with Matlab software. Consider a symmetric case, where the accuracy of the support algorithm (*q*) is similar for both device types ($q_{H}=q_{L}=q$). We assume that the probability to receive adjusted support depends on the accuracy level of the device’s support algorithm; for high privacy risk devices it is two times more probable than for low privacy risk devices, i.e., $P_{a}\left(q,H\right)=q$ and $P_{a}\left(q,L\right)=0.5q$. We further assume the following parameters are given as input: π=0.5 (equal prior to each type), $u_{1}=v_{1}=1$, $u_{L}=1$, $u_{H}=2$, $E_{L}^{2}=2$; $E_{H}^{2}=4$, $u_{b}=-3$, $P_{b}\left(L\right)=0.2$ and $P_{b}\left(H\right)=0.8$.

Note that these parameter values were intentionally chosen. With these values, if the device is of H-type, the expected utility of the user at stage 2 is negative: $\left(1-P_{b}\left(H\right)\right)u_{H}+P_{b}\left(H\right)u_{b}=-2$. If the device is of L-type, the expected utility of the user at stage 2 is positive: $\left(1-P_{b}\left(L\right)\right)u_{L}+P_{b}\left(L\right)u_{b}=0.2$. Thus, these values present a non-trivial setting where it is unclear what the user should do. In contrast, when the expected user utility is negative for both high and low device types, the user has no incentive to use the device and will abandon it. Similarly, when the user-expected utility is positive for both device types, the user will always use the device regardless of its type. Thus, the values were specifically chosen to accommodate the non-trivial case were the expected value is negative for one device type and positive for the other device type.

We performed the following procedure for different values of *q* ranging from 0 to 1. First we randomly generated two Bernoulli trials:

1. A Bernoulli trial for a signal on the device type s∈{h,l}.
2. A Bernoulli trial for the event that adjusted support is sent or not sent to the user $e\in \{a,\bar{a}\}$.

When the signal is *a* the device utility is $v_{1}$.

Then, we calculated the user belief of the device type (according to (A1)–(A4)). Next we calculated whether the user prefers to continue using the device in stage 2 (according to (A5)). If the user stays, the utility increases in $E_{H}^{2}$ and $E_{L}^{2}$ for devices with high (*H*) and low (*L*) privacy risks, respectively.

For each q∈{0,0.1,...,1} we ran this procedure for 10,000 trials, and then computed the average total utility (profit) of high and low-risk device types.

**Figure 1:**
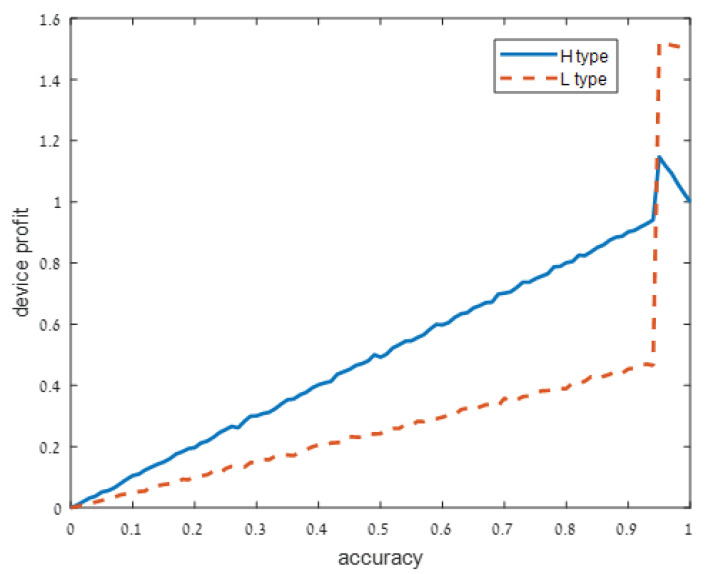
Extremely noisy signal: ϵ=0.49.

**Figure 2:**
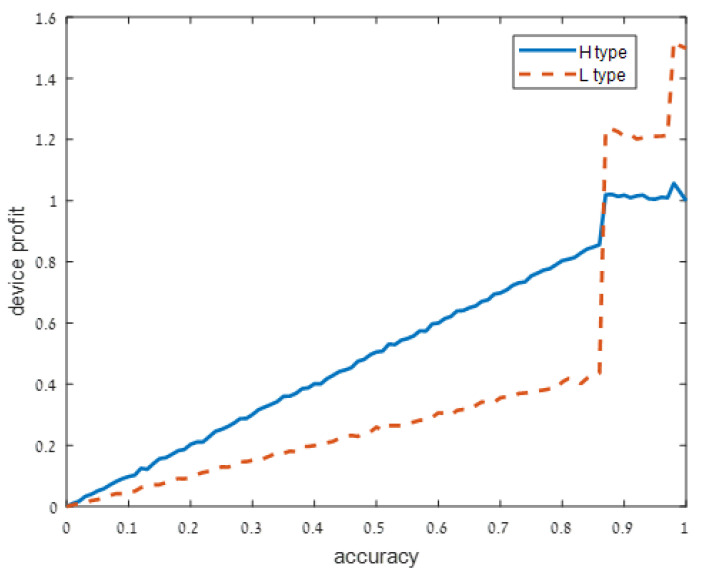
Moderately noisy signal: ϵ=0.3.

**Figure 3:**
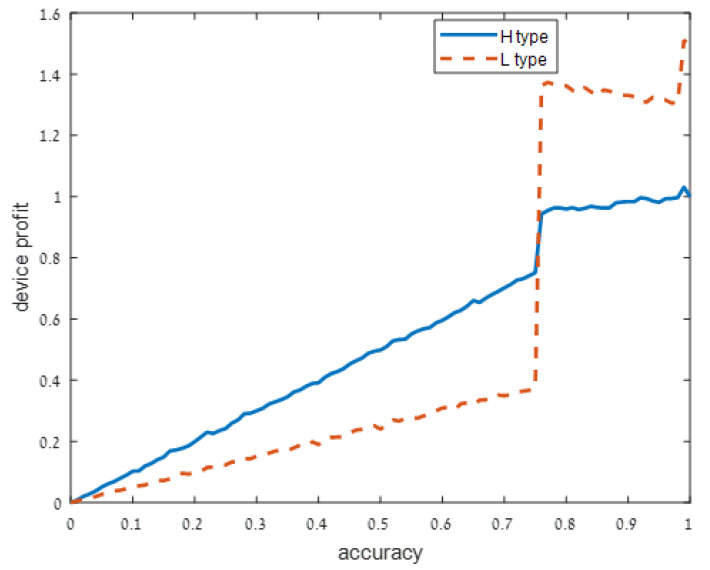
Moderately noisy signal: ϵ=0.2.

**Figure 4:**
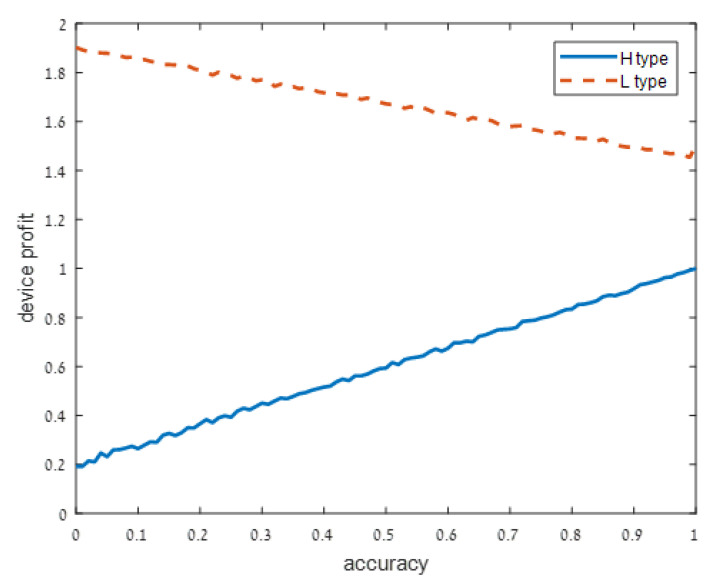
Low noise: ϵ=0.05.

We present our numerical results in Figure 1, Figure 2, Figure 3 and Figure 4. Each figure considers one of the following four signal accuracy levels ϵ∈{0.49,0.3,0.2,0.05}. Recall that a lower ϵ means that the user has a better understanding of what the privacy risk is (at ϵ=0 the user knows the risk for certain). The figures illustrate the average total profit (axis *y*) as *q*, the accuracy of the support algorithm (axis *x*) increases.

Figure 1 shows that when the signal on the device type is extremely noisy (ϵ=0.49), both high and low device types profit from a higher accuracy strategy *q*, but the high-risk device type is better off if q=0.9. In other words, both device types profit from sharing more accurate information and support with the user, but high privacy risk devices should be careful not to fully utilize all of their capabilities.

As user understanding of the privacy increases, i.e., as ϵ decreases from ϵ=0.49 in Figure 1 to ϵ=0.3 and ϵ=0.2 in Figure 2 and Figure 3, respectively, the devices with a high privacy risk (type H) maximize their profit at lower accuracy values. This does not hold when the user knowledge of the device’s risk is high (ϵ=0.05 in Figure 4), since in this case, the users are aware of the privacy risk and thus the profit is maximized when the device outputs accurate support.

When the noise ϵ is low, users know with high probability the type of device. It is not surprising then that the utility of the high-type device increases in *q*. It is more surprising that the utility of the low type device decreases in *q*. This may be explained by the fact that a negative effect of the adjusted support still persists (there is a small, but positive probability that following signal *a* the user will suspect that the device is of high risk). However, this effect is relatively weak.

## 5. Discussion

We analyzed how privacy risk considerations affect the decisions of both the devices and the users. Our main finding herein is that when the device’s privacy risk is unknown to the users it might be inefficient for the device to exploit its sensing technology, since this may lead users to abandon the device.

Specifically, based on our three analytical propositions, we suggest the following for optimal acceptance of devices provided with sensors when the privacy risk is unknown to the users. Our suggestions focus on the communication between the device and the user, specifically on the device feedback policy. These suggestions do not depend on technical characteristics of device, e.g., the sensor’s accuracy and cost.

1. **Assure low risk**—Convincing the user that the device has a low privacy risk for them is of urgent importance. Risk-concerned users who are not convinced will quickly abandon the device. For example, publish a clear and easy to understand privacy policy.
2. **Limit initial accurate feedback**—Accurate advice and assistance might be the most intuitive way to exhibit the device’s usefulness. However, at the first usage period the device should do so with caution. A risk-concerned user might abandon the device due to accurate feedback. So this feedback should sometimes be withheld. This is because, from accurate feedback, the user concludes that their privacy is being compromised.
3. **Second stage accurate feedback is welcome**—Once the user is aware of the privacy risk, and given that they did not abandon the device in the first usage period, they will probably not abandon the device due to privacy concerns later on. If users do not identify a risk, they will keep using the device.

Each of these propositions is both grounded analytically, and also intuitive to understand. However, applying the three of them in practice is not trivial. In future work we plan to apply these recommendations to a wearable device.

## Figures and Tables

**Table 1:** List of notations in the model.

Notation	Description
*S*	device
*C*	user
ρ	device type (H-high risk; L-low risk)
π	prior probability of high risk
*s*	signal about device type
ϵ	noise of the signal the user receives about device type
*q*	accuracy level of device support algorithm
$q_{H}$	accuracy level of high risk device support algorithm
$q_{L}$	accuracy level of low risk device support algorithm
$P_{a}\left(q,\rho \right)$	probability to receive adjusted support
*a*,$\bar{a}$	events “support sent/not sent to the user”
P(H|s,e)	probability assigned by the user to the device being H
$P_{b}\left(\rho \right)$	probability of private information leaked
$u_{1}$	user utility from the adjusted support
$v_{1}$	device utility from the adjusted support
$u_{b}$	user utility if private information is leaked
$v_{b}$	device utility if private information is leaked
$u_{H}$	user utility at stage 2 from the high risk device, if no information is leaked
$u_{L}$	user’s utility at stage 2 from the low risk device, if no information is leaked
$E_{H}^{2}$	high-risk device utility at stage 2
$E_{L}^{2}$	low-risk device utility at stage 2

## Appendix A

**Proof** **of Proposition 1.** Following signal *h* and event *a*, C’s belief that S is H is

(A1) $$P\left(H|h,a\right)=\frac{\pi \left(1-\epsilon \right)P_{a}\left(q_{h},H\right)}{\pi \left(1-\epsilon \right)P_{a}\left(q_{h},H\right)+\left(1-\pi \right)\epsilon P_{a}\left(q_{L},L\right)}.$$

Following signal *h* and event $\bar{a}$, C’s belief that S is H is

(A2) $$P\left(H|h,\bar{a}\right)=\frac{\pi \left(1-\epsilon \right)(1-P_{a}\left(q_{h},H\right))}{\pi \left(1-\epsilon \right)\left(1-P_{a}\left(q_{h},H\right)\right)+\left(1-\pi \right)\epsilon \left(1-P_{a}\left(q_{L},L\right)\right)}.$$

Following signal *l* and event *a*, C’s belief that S is H is

(A3) $$P\left(H|l,a\right)=\frac{\pi \epsilon P_{a}\left(q_{h},H\right)}{\pi \epsilon P_{a}\left(q_{h},H\right)+\left(1-\pi \right)\left(1-\epsilon \right)P_{a}\left(q_{L},L\right)}.$$

Following signal *l* and event $\bar{a}$, C’s belief that S is H is

(A4) $$P\left(H|l,\bar{a}\right)=\frac{\pi \epsilon (1-P_{a}\left(q_{h},H\right))}{\pi \epsilon \left(1-P_{a}\left(q_{h},H\right)\right)+\left(1-\pi \right)\left(1-\epsilon \right)\left(1-P_{a}\left(q_{L},L\right)\right)}.$$

Let s∈{l,h} be a signal received by C about S’s type, and $e\in \{a,\bar{a}\}$ be the event outcome of stage 1. C prefers not to leave at stage 2 iff

(A5) $$P\left(H|s,e\right)\left[P_{b}\left(H\right)u_{b}+\left(1-P_{b}\left(H\right)\right)u_{H}\right]+\left(1-P\left(H|s,e\right)\right)\left[P_{b}\left(L\right)u_{b}+\left(1-P_{b}\left(L\right)\right)u_{L}\right]\geq 0,$$

which is equivalent to

(A6) $$-u_{b}\leq \frac{P\left(H|s,e\right)\left[\left(1-P_{b}\left(H\right)\right)u_{H}-\left(1-P_{b}\left(L\right)\right)u_{L}\right]+\left(1-P_{b}\left(L\right)\right)u_{L}}{P\left(H|s,e\right)\left[P_{b}\left(H\right)-P_{b}\left(L\right)\right]+P_{b}\left(L\right)}.$$

Let $u_{H}>\frac{P_{b}\left(H\right)u_{L}\left(1-P_{b}\left(L\right)\right)}{\left(1-P_{b}\left(H\right)\right)P_{b}\left(L\right)}$. Then the right-hand side of (A6) increases in P(H|s,e) and its maximum, obtained for P(H|s,e)=1, is

$$\frac{\left(1-P_{b}\left(H\right)\right)u_{H}}{P_{b}\left(H\right)},$$

and the minimum (at P(H|s,e)=0) is

$$\frac{\left(1-P_{b}\left(L\right)\right)u_{L}}{P_{b}\left(L\right)}.$$

Therefore, for $\frac{\left(1-P_{b}\left(H\right)\right)u_{H}}{P_{b}\left(H\right)}<-u_{b}$, C prefers to leave at stage 2 for any P(H|s,e). For $-u_{b}<\frac{\left(1-P_{b}\left(L\right)\right)u_{L}}{P_{b}\left(L\right)}$, C prefers not to leave at stage 2 for any P(H|s,e). If $\frac{\left(1-P_{b}\left(L\right)\right)u_{L}}{P_{b}\left(L\right)}<-u_{b}<\frac{\left(1-P_{b}\left(H\right)\right)u_{H}}{P_{b}\left(H\right)}$, there exists $P_{H}^{*}$ such that (A6) holds iff $P_{H}^{*}\leq P\left(H|s,e\right)$. Next, suppose $u_{H}<\frac{P_{b}\left(H\right)u_{L}\left(1-P_{b}\left(L\right)\right)}{\left(1-P_{b}\left(H\right)\right)P_{b}\left(L\right)}$. Then the right-hand side of (A6) decreases in P(H|s,e). For $\frac{\left(1-P_{b}\left(H\right)\right)u_{H}}{P_{b}\left(H\right)}>-u_{b}$, C prefers not to leave at stage 2 for any P(H|s,e). For $-u_{b}>\frac{\left(1-P_{b}\left(L\right)\right)u_{L}}{P_{b}\left(L\right)}$, C prefers to leave for any P(H|s,e). If $\frac{\left(1-P_{b}\left(L\right)\right)u_{L}}{P_{b}\left(L\right)}>-u_{b}>\frac{\left(1-P_{b}\left(H\right)\right)u_{H}}{P_{b}\left(H\right)}$, there exists $P_{H}^{*}$ such that (A6) holds iff $P_{H}^{*}\geq P\left(H|s,e\right)$.  □

**Proof** **of Proposition 2.** Suppose by contrary $q_{H}=q_{L}=1$ in equilibrium. By assumption $P_{a}\left(1,H\right)\left[1-P_{a}\left(1,L\right)\right]>P_{a}\left(1,L\right)\left[1-P_{a}\left(1,H\right)\right]$, thus by continuity there is $\epsilon <\frac{1}{2}$ such that $\epsilon^{2}P_{a}\left(1,H\right)\left[1-P_{a}\left(1,L\right)\right]>{\left(1-\epsilon \right)}^{2}P_{a}\left(1,L\right)\left[1-P_{a}\left(1,H\right)\right]$, and by (A2) and (A3), $P\left(H|h,\bar{a}\right)<P\left(H|l,a\right)$. By (A1)–(A4), for $\epsilon <\frac{1}{2}$, P(H|h,e)>P(H|l,e), $s=a,\bar{a}$. To summarize,

(A7) $$P\left(H|l,\bar{a}\right)<P\left(H|h,\bar{a}\right)<P\left(H|l,a\right)<P\left(H|h,a\right).$$

Let RHS(P(H|s,e)) be the right-hand side of (A6) for given P(H|s,e). For $u_{H}<\frac{P_{b}\left(H\right)u_{L}\left(1-P_{b}\left(L\right)\right)}{\left(1-P_{b}\left(H\right)\right)P_{b}\left(L\right)}$, RHS(P(H|s,e)) decreases in P(H|s,e)). Therefore,

(A8) $$RHS\left(P\left(H|l,\bar{a}\right)\right)>RHS\left(P\left(H|h,\bar{a}\right)\right)>RHS\left(P\left(H|l,a\right)\right)>RHS\left(P\left(H|h,a\right)\right).$$

By (A6) and by (A8), if $RHS\left(P\left(H|l,a\right)\right)<|u_{b}|<RHS\left(P\left(H|h,\bar{a}\right)\right)$, for both *h* and *l* signals C chooses not to leave following $\bar{a}$ and to leave following *a*. The expected utility of *H* is therefore

$$P_{a}\left(1,H\right)v_{1}+\left(1-P_{a}\left(1,H\right)\right)E_{H}^{2},$$

and by $v_{1}<E_{H}^{2}$, and by being $P_{a}\left(q_{H},H\right)$ decreasing in $q_{H}$, *H* is better off by deviating to $q_{H}<1$. Similarly, *L* is better off by deviating to $q_{L}<1$, contradiction.  □

**Proof** **of Proposition 3.** By (A1)–(A4), for every $e\in \{a,\bar{a}\}$, P(H|h,e)→1 and P(H|l,e)→0 as ϵ→0. Therefore, the right-hand side of (A6) converges to a constant independent on *a* or $\bar{a}$, and C chooses to leave or not to leave at stage 2, independent on even *a* or $\bar{a}$. Thus, both *H* and *L* maximize their payoffs at stage 1, which are maximized for $q_{H}=q_{L}=1$.  □
